# A Review of Eptinezumab Use in Migraine

**DOI:** 10.7759/cureus.18032

**Published:** 2021-09-16

**Authors:** Abhigyan Datta, Shashi Maryala, Rebecca John

**Affiliations:** 1 Medicine, Maulana Azad Medical College, New Delhi, IND; 2 Neurology, Gandhi Hospital, Hyderabad, IND; 3 Neurology, Kannur Medical College, Kannur, IND

**Keywords:** safety of eptinezumab, efficacy of eptinezumab, clinical pharmacology, anti-cgrp monoclonal antibody, review of clinical trials, migraine disorder, eptinezumab

## Abstract

We present a review of the efficacy and safety profile of eptinezumab (also known by the brand name Vyepti), a calcitonin gene-related peptide (CGRP) monoclonal antibody (mAb) developed by Lundbeck Seattle BioPharmaceuticals, Inc., that received its first approval in the USA on 21 February 2020 for the preventive treatment of migraine in adults. It is administered by an intravenous infusion at a 100 mg dose every 3 months and shows no drug interactions.

Studies have shown that eptinezumab is an effective preventative medication in migraine which starts showing its effect from day 1 of its administration, which maintains a consistent level of efficacy through a year of its treatment at doses 100 mg and 300 mg. It was found to be effective at reducing time to headache pain freedom during acute migraine attacks as well.

Eptinezumab is a relatively safe drug for the prevention of migraines with treatment-related adverse events occurring at a low frequency. They bear a safe profile in patients with comorbidities like obesity and type 1 diabetes. The most frequent adverse events observed were nasopharyngitis, upper respiratory tract infections (URTIs), and sinusitis and were usually mild. The development of anti-drug antibodies was common, but they declined to undetectable levels with continued dosing and did not appear to impact the overall safety profile of the drug. Further studies are needed to assess long-term safety, use in different patient populations, and to compare its efficacy to other drugs of its class.

## Introduction and background

According to the International Classification of Headache Disorders, migraine is defined as a recurrent headache disorder manifesting in attacks lasting 4-72 hours [1]. In the Global Burden of Disease 2019, migraine ranks second among the world’s causes of disability, and first among young women between the ages of 15 and 49 [2]. Over the past few years, monoclonal antibodies (mAb) specifically targeting calcitonin gene-related peptide (CGRP) molecules have shown an incredible effect as a preventative migraine medication [3,4]. While not fully understood, these are thought to block CGRP molecules that help conduct the pain signals from the trigeminal ganglion into the higher centers of the brain [5-7].

One such antibody is eptinezumab (also known by the brand name Vyepti) [8]. Developed by Lundbeck Seattle BioPharmaceuticals, Inc., eptinezumab received its first approval in the USA on 21 February 2020 for the preventive treatment of migraine in adults [9]. Eptinezumab is administered by intravenous (IV) infusion at a dosage of 100 mg every 3 months [8]. This makes it the first intravenous anti-CGRP monoclonal antibody, with the other three FDA-approved drugs of the same class (erenumab, fremanezumab, and galcanezumab) being administered subcutaneously [10-12]. In this review, we discuss the pharmacology, efficacy, safety, and future of this novel drug. 

## Review

Search strategy

To review the use of eptinezumab for migraine, a PubMed and Cochrane search using the keyword “Eptinezumab” and “migraine” was made in August 2021, which yielded 84 articles. We also looked upon the references of these articles and the final bibliography was established accordingly.

Pharmacology

**Figure 1:**
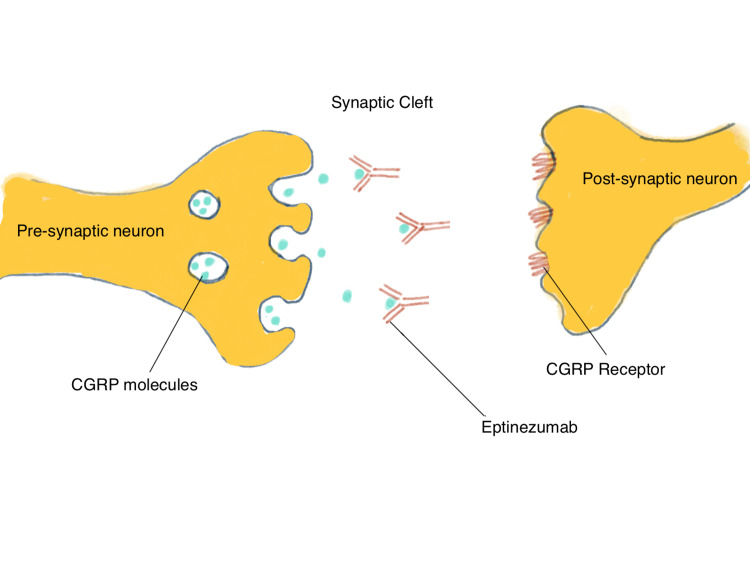
Pictorial representation of the mechanism of action of eptinezumab The above figure shows the blockage of calcitonin gene-related peptide (CGRP)molecules by eptinezumab from binding to the CGRP receptor.

Eptinezumab is a humanized IgG1 monoclonal antibody produced by recombinant DNA techniques within yeast cells of *Pichia pastoris *[8] that has been in the market for approximately one year for the prevention of migraines. During a migraine, the trigeminal nerve conducts the pain signal via CGRP into the brainstem and to higher order regions of the brain [5-7]. Thus, eptinezumab is hypothesized to prevent migraines by binding to (and blocking) CGRP molecules. It can specifically and rapidly bind to both α- and β-CGRP ligands to block it from binding to CGRP receptors (Figure 1), however, it is slow to dissociate, which might explain its rapid onset and longer duration of effect [9]. 

Eptinezumab is the only drug of its class to be administered intravenously, and steady-state plasma concentrations are achieved after the first dose [8]. Studies of the antibody have shown that its bioavailability is 100% by the end of its half-hour infusion [13]. It is not metabolized by cytochrome P450 enzymes, reducing drug-drug interactions, and one study showed that its pharmacokinetics were not significantly affected by coadministration of 6mg subcutaneous sumatriptan [8].

It has a half-life of about 28 days, with a central volume of distribution of around 3.7 L and total plasma clearance of 0.125 l/day [9]. A recent study combined the results of eight pharmacokinetics studies and found that the maximum serum concentration (Cmax) after single 100 mg and 300 mg doses were 37.3 and 114 μg ml-1 respectively and the Tmax was 30 minutes-1 hour, which might explain its rapid onset [14]. This was not significantly affected by patient factors such as age, sex, race, body weight, and whether they were suffering from migraine or controlled [14]. No studies have been performed on its use during pregnancy.

Efficacy

Phase 2 Trials

Two Phase 2 trials were conducted in 2014 and 2019 to evaluate the safety and clinical efficacy of eptinezumab [15,16]. The first trial (NCT01772524) was a proof-of-concept trial and was designed as a randomized, double-blind, placebo-controlled Phase 2 trial in which 174 patients aged 18-55 years coming from 26 US centers were randomly assigned a single 1000 mg intravenous infusion of eptinezumab or placebo [15]. The primary objective was to assess safety at 3 months after the infusion while the primary efficacy endpoint was a change in Monthly Migraine Days (MMDs) from baseline at 5-8 weeks. The study found a decrease in MMDs by 5.6 days for eptinezumab compared to 4.6 days for placebo (p = 0.0306). Upper respiratory tract infections (seen in 9% of patients) were the most reported adverse event in the eptinezumab group. Other adverse events included tooth abscess, nausea and vomiting, dizziness, fatigue, and QTcF prolongation. This study paved the way for future trials of this medication.

Another trial (NCT02275117) was conducted in 2019 to assess the effectiveness of 300, 100, 30, and 10 mg doses of intravenous eptinezumab [16]. This was a randomized, placebo-controlled, double-blind trial with the parallel design of 588 patients with chronic migraine (≥14 MMDs a month) who had migraine onset at ≤35 years, conducted in 92 international sites. The primary endpoint was a reduction of 75% or more in migraine days over weeks 1-12, as compared with the 28-day screening period. The percentage of responders for 300, 100, 30, and 10 mg were 33.3, 31.4, 28.2, and 26.8%, respectively, compared to 20.7% for placebo, but only the 300 mg group was statistically significant (p = 0.033). The most frequent adverse events were upper respiratory tract infection and dizziness.

Phase 3 Trials - PROMISE‑1 and PROMISE‑2 

PROMISE-1 (NCT02559895) was a randomized, double-blind, multicentre, placebo-controlled study in 888 adults (aged 18-75 years) with a history of episodic migraine (4-14 monthly migraine days) for ≥ 12 months [17]. These patients received eptinezumab 30, 100, or 300 mg, or placebo once every 3 months for 60 weeks. Patients were allowed to use other acute migraine medications such as triptans. The primary endpoint of the study was a change in MMDs over weeks 1-12 from baseline. Migraines were recorded using an electronic diary daily (eDiary) and clinic visits were scheduled. The 12-week primary study found a reduction of MMDs by 3.9 (p = 0.0182) and 4.3 (p < 0.0001) days between 100 mg and 300 mg eptinezumab groups respectively in comparison to placebo (3.2 days). In addition, 31% (p = 0.01) and 32% (p < 0.01) patients in the 100 mg and 300 mg groups respectively had ≥ 75% reduction from baseline in MMDs over weeks 1-4 compared to placebo.

Smith et al reported results of the above PROMISE -1 trial after one year of treatment and concluded that eptinezumab provides sustained migraine-preventive effects and an acceptable tolerability profile [18]. Interestingly, the study found that the proportions of patients experiencing ≥50% and ≥75% migraine response were greater during weeks 24-48 compared to weeks 1-24 of the study. Thus, a longer duration of treatment beyond the initial 12 weeks provides further benefit. As detailed further in the next section, the drug was also found to be safe with few adverse effects over 1 year of treatment.

Similar results were reported in the PROMISE-2 (NCT02974153) study, a randomized, double-blind, multicentre, placebo-controlled trial that involved 1072 adults with chronic migraine [19]. These patients were divided into three groups: eptinezumab 100 mg (n=356) or 300 mg (n=350) or placebo (n=366) every 3 months for 6 months; and they were allowed to take other migraine preventatives (except onabotulinumtoxinA). The primary study (over the first 12 weeks) reported significantly reduced migraine frequency at the end of week 12 (which was the primary endpoint), with a reduction in MMD of 7.7 days in the 100 mg group, and−8.2 days in the 300 mg group vs−5.6 days in placebo (p ≤ 0.0001 for both groups). In terms of responder rates, 27% and 33% of 100 mg and 300 mg groups respectively had ≥75% responder rates vs 15% for the placebo group. These benefits were sustained till the end of the 24 week treatment period with no new side effects [20]. The ≥50% and ≥ 75% migraine responder rates (MRRs) increased after a second dose, with more eptinezumab-treated patients experiencing migraine response than placebo patients [20].

Subsequent post hoc analysis of the PROMISE-2 trial revealed that within the first 4 weeks itself, eptinezumab 100 or 300 mg increased the number of days free from typical migraine symptoms such as nausea, vomiting, and photophobia; improved ratings on the patient-reported patient global impression of change (PGIC) scale (45% and 59% vs 32%), and significantly reduced the functional impact of headache (assessed by the six-item Headache Impact Test or HIT-6 total score) with this benefit sustained even at 24 weeks [21].

This benefit was seen even in the subgroup of patients in PROMISE-2 who very often or always experienced severe pain during headaches [22]. In this subgroup, both eptinezumab 100 or 300 mg over 12 weeks improved the frequency of severe headaches (decrease in severity by at least 1 category), the patient reported disease status (assessed by the PGIC scale), the frequency of migraine (reduction in MMDs), health-related quality of life [assessed by the Short-Form Health Survey] and daily functioning (assessed by HIT-6) [22]. In terms of health-related quality of life (HRQoL), one interesting finding of a post hoc analysis of both the PROMISE-1 and PROMISE-2 trials was that the improvements following eptinezumab administration were greater in patients with higher MMDs at baseline [23]. Thus, the beneficial effects of this drug seem to be greater in patients with a more severe migraine.

Subgroup analysis also revealed that the reduction in MMDs was seen regardless of patient factors such as age, race, gender, migraine duration, baseline MMDs, and concurrent use of triptans or preventive medication [9]. Other post hoc analyses of the abovementioned trials also demonstrated that 63% in the eptinezumab 100 mg group and 64% in the eptinezumab 300 mg group (vs 48% in the placebo group) had ≥ 1 migraine-free month in the PROMISE-1 trial and 35% in the eptinezumab 100mg group and 40% in the eptinezumab 300 mg group (vs 22% in the placebo group) had ≥ 1 migraine-free month in the PROMISE-2 trial [24]. 

Diener et al conducted another subgroup analysis of the PROMISE-2 trial to evaluate eptinezumab 100 mg and 300 mg in patients having both diagnoses of chronic migraine (CM) and medication-overuse headache (MOH) [25]. They found that eptinezumab treatment resulted in greater reductions in MMDs, higher responder rates, and fewer patients meeting CM and MOH criteria post-treatment, thus highlighting the efficacy of eptinezumab in this patient population.

The above studies highlighted the efficacy of eptinezumab as a preventative migraine medication but did not explore the benefits of its rapid onset of action, which is one of the most exciting findings of this drug. ≥ 50% of patients with episodic migraine in PROMISE-1 and chronic migraine in PROMISE-2 experienced a significant reduction in MMDs within the first 4 weeks of treatment [26]. In fact, the positive effects were seen even on the first day in both trials, with the proportion of patients with migraine one day after the infusion being significantly less in both the eptinezumab 100 mg (14.8%, p < 0.05) and 300 mg (13.9%, p < 0.05) groups compared with placebo (22.5%) in the PROMISE-1 trial [27]. Eptinezumab was also shown to reduce the use of concurrent abortive medication compared to placebo [28].

PREVAIL Trial

PREVAIL was a recent open-label, Phase 3 trial comprising of two 48-week treatment phases in 128 adults with chronic migraine for ≥12 months (aged 18-65 years, diagnosed with a chronic migraine before the age of 50), who received intravenous eptinezumab 300 mg once every 12 weeks (a total of eight doses) [29]. Unlike previous trials, this study was primarily done to assess the long-term safety, immunogenicity, and tolerability of eptinezumab. Treatment-emergent adverse events were seen in 65% of patients; these are described in greater detail below. 

In addition, the study also corroborated the findings of the previous trials, as eptinezumab 300 mg was associated with improved patient-reported outcome measures assessed by the Migraine Disability Assessment or MIDAS questionnaire, patient-identified most bothersome symptom (MBS) associated with migraine, PGIC, and HIT-6 [29]. 

Miscellaneous Trials

Apart from these three landmark trials, other trials have also been performed to determine the possible use of eptinezumab in different circumstances. Recently, Winner et al conducted the RELIEF study, which is the first study that evaluated the efficacy of eptinezumab during an acute migraine attack [30]. This was a randomized, double-blinded placebo-controlled multicenter study identifying the efficacy and adverse events of administering 100 mg eptinezumab in 480 adults with a history of migraine ≥12 months during a moderate to severe migraine attack. The primary endpoint of the trial was to observe for the time to headache pain freedom and time to the absence of the most bothersome symptoms (nausea, photophobia, phonophobia). The study found that patients on eptinezumab showed statistical significance in reduction of time to headache pain freedom (median, 4 hours with eptinezumab vs 9 hours on placebo; Hazard ratio 1.54 [p < 0.001]) and a reduction in time to the absence of the most bothersome symptom (median, 2 hours with eptinezumab vs 3 hours on placebo; Hazard ratio 1.75 [p<0.001]). The most common treatment-emergent adverse event was hypersensitivity (2.9% on eptinezumab vs 0% on placebo). Although eptinezumab shows promise here, further studies are needed to evaluate its feasibility in such a setting, given its intravenous route of administration.

Baker et al. conducted two randomized, double-blind, placebo-controlled trials to assess the safety profile and metabolic effects of eptinezumab in 24 patients with obesity/overweight (study 1), assessed by changes in basal metabolic rate, as well as 21 patients with type 1 diabetes or T1D (study 2), assessed by insulin sensitivity in terms of body weight and insulin concentration corrected glucose infusion rate (M/I) [31]. Study 1 found no significant difference in basal metabolic rate (BMR)** **between eptinezumab 100 mg and placebo at 7 days post‐administration (least-squares mean change in BMR was 31.6% [95% CI -90.6, 153.8] which was not statistically significant). Study 2 also showed no significant between‐group differences in various measures of insulin sensitivity between eptinezumab and placebo groups (changes in M/I ratios, change from baseline to day 7 in the body weight‐corrected glucose infusion rate (GIR) or the insulin sensitivity index). Both the studies had similar rates of adverse events between the eptinezumab and placebo groups. Thus, the study found that it is safe to administer eptinezumab in patients with T1D or those who were overweight/obese.

Adverse events

Both the PROMISE-1 and PROMISE-2 trials showed that eptinezumab was well tolerated and safe. Nasopharyngitis was the most commonly reported adverse event (AE) [8]. In the PROMISE-1 trial, treatment-emergent adverse events (TEAEs) (incidence>5% and greater than placebo) in the eptinezumab 30 mg, 100 mg, 300 mg and placebo groups, included viral upper respiratory tract infection (11.4%, 9,9% and 10.3% respectively compared to 7.2% in placebo group) and nasopharyngitis (6.4%, 7.6% and 6.3% respectively compared to 5.4% in placebo group) [17,18]. Out of these, the most commonly reported study-drug-related TEAEs were nausea (n = 14 [1.6%]) and fatigue (n = 12 [1.4%]). TEAEs were usually mild or moderate in severity, with severe TEAEs occurring in 2% of eptinezumab groups and 3% of placebo groups. None of these serious events were considered related to the drug. These led to treatment discontinuation in 6%, 3%, 2% of patients receiving eptinezumab 30, 100 or 300 mg (compared to 3% of placebo recipients). Similar findings were also reported in the PROMISE-2 study, except that no treatment-related serious AEs were reported [19,20]. 

In the PREVAIL trial, at least one TEAE was seen in 71% of eptinezumab recipients; the most commonly reported effects were nasopharyngitis (14.1%), upper respiratory tract infection (7.8%) sinusitis (7.8%), and influenza (6.3%), and were usually mild to moderate in severity [29]. 18 patients (14.1%) had at least one TEAE that was considered related to the study drug. These included hypersensitivity and nausea that were reported in 4% and 3% of recipients respectively. Notably, the drug was discontinued in 6.3% of patients due to adverse events. There were three pregnancies during this trial, out of which two ended prematurely (due to miscarriage or termination) and the third was a full-term delivery.

Smith et al. performed a pooled analysis of 4 randomized double-blinded, placebo-controlled trials (PROMISE1, PROMISE2, NCT01772524, NCT02275117) and the first year of the PREVAIL trial to determine the safety profile of eptinezumab as compared to a placebo [32]. TEAEs were graded on a scale of 1 to 5, 1 being a mild event and 5 being death. About 54.8% of patients on eptinezumab and 52.3% of patients on placebo experienced 1 or more TEAEs. Of these, 14.2 % on eptinezumab and 9.4% on placebo were considered as treatment-related AEs. Thirty infusion-site AEs occurred in 27/2076 (1.3%) patients who received eptinezumab and 7 in 7/791 (0.9%) patients who received placebo. Hypersensitivity occurred in 23/2076 (1.1%) patients treated with eptinezumab and no patients in the placebo group. Most hypersensitivity reactions were not serious and resolved with standard medical treatment or observation without treatment, usually within 1 day. 

One of the potential risks with anti-CGRP monoclonal antibodies is the risk of immunogenicity [8]. All of the above trials also reported the presence of anti-eptinezumab antibodies. 21%, 18% and 18% of eptinezumab recipients developed anti-eptinezumab antibodies in the PROMISE-1, PROMISE-2, and PREVAIL trials, and out of these, 41%, 35%, and 39% developed anti-eptinezumab neutralizing antibodies, respectively [17,19,29]. It is difficult to assess the clinical impact of the development of these anti-eptinezumab antibodies, however, the above studies reported no impact of the development of these antibodies on the safety profile of eptinezumab [8,29]. Overall, the drug appears to be very safe with few adverse effects. A summary of the various trials with adverse effects has been illustrated in Table 1 [17, 19, 29, 30].

**Table 1:** A synopsis and comparisons describing the eptinezumab trials MMD - Monthly Migraine Days, URTI - Upper respiratory tract infections, TEAEs -Treatment-emergent adverse event, MIDAS - Migraine disability assessment test, PGIC - Patient Global Impressions Scale - Change, HIT-6 - Headache Impact Test

Study	Inclusion criteria	Dose, frequency, administration	Primary Endpoint	Results	Common adverse events
PROMISE-1 [17]	Adults with 4-14 monthly migraine days (MMDs) for ≥ 12 months	Intravenous Eptinezumab 30, 100 or 300 mg or placebo once every 3 months for 60 weeks	Change in MMDs over weeks 1-12 from baseline	Reduction of MMDs by 3.9 (p=0.0182) and 4.3 (p<0.0001) days between 100mg and 300mg eptinezumab groups respectively in comparison to placebo (3.2 days)	URTIs, nasopharyngitis
PROMISE-2 [19]	Adults 18 to 65 years of age with chronic migraine at or before 50 years of age for ≥12 months	Intravenous Eptinezumab 100 mg or 300 mg or placebo every 3 months for 6 months	Change from baseline in MMDs over weeks 1 to 12, assessed with eDiary data.	Reduction in MMD of 7.7 days in the 100mg group, and−8.2 days in the 300mg group vs−5.6 days in placebo (p≤0.0001)	Nasopharyngitis, URTIs, nausea
PREVAIL [29]	Adults with chronic migraine for ≥12 months (aged 18-65 years, diagnosed with a chronic migraine before the age of 50)	Intravenous Eptinezumab 300 mg once every 12 weeks (a total of 8 doses)	To assess safety (by monitoring of TEAEs), immunogenicity (development of anti-drug antibodies), and patient-reported outcomes (MIDAS questionnaire, PGIC, HIT-6).	Nasopharyngitis (14%) and URTI (8%) were the most frequent adverse events. The drug was discontinued in 6.3% of patients due to adverse events. Anti Drug antibodies peaked at week 24 and declined to nondetectable levels at week 104 despite continued dosing. The patient reported outcomes improved at first assessment and were sustained throughout.	Nasopharyngitis, URTIs, sinusitis, and influenza.
RELIEF [30]	Adults with a history of migraine ≥12 months during a moderate to a severe migraine attack	Intravenous eptinezumab 100mg vs placebo within 1-6 hours of moderate to a severe migraine attack	Time to headache pain freedom and time to the absence of the most bothersome symptoms (nausea, photophobia, phonophobia)	Faster headache pain freedom (median, 4 hours on eptinezumab vs 9 hours on placebo; hazard ratio, 1.54) as well as the absence of most bothersome symptom (median, 2 hours on eptinezumab vs 3 hours on placebo; hazard ratio, 1.75).	Hypersensitivity

Future of eptinezumab

Eptinezumab appears to have a very favorable safety and efficacy profile and is certainly a welcome addition to the ever-growing arsenal of preventative migraine medications. However, further studies need to be conducted over a longer time frame of 3-5 years to assess long-term safety. Other drugs of its class, such as erenumab and fremanezumab, having received approval earlier, are more proven in this aspect [33,34]. 

In addition, studies are also required to test its safety in specific subgroups, such as pregnant patients and children. NCT04537429 is a clinical trial with 32 participants aged 6-17 years to evaluate the pharmacokinetic properties of eptinezumab in this age group, with a secondary aim to track the development of anti-eptinezumab antibodies [35]. Another trial, NCT04965675 aims to evaluate the efficacy and safety of eptinezumab in adolescents aged 12-17 [36]. Most of the trials so far have been conducted in the United States, and little is known about the possible effects of this drug in other patient populations. NCT04336449 is a trial conducted on 18 Japanese patients investigating the tolerability, safety, and pharmacokinetic properties of eptinezumab and was recently completed, although the results have not been published yet [37]. No comparative studies have been performed between eptinezumab and other preventive medications as well.

The success of eptinezumab in real-world scenarios is still yet to be determined. While randomized controlled trials have highlighted its efficacy and safety, they can have limitations and may not represent the patient population seen in the clinic on a routine basis. Additional research is needed to evaluate the specific patient characteristics for which eptinezumab will be most useful. The cost for each infusion every 3 months is around $1600, but the total yearly cost is comparable to the price of using the other anti-CGRP monoclonal antibodies for a year [38].

## Conclusions

In this review, we looked at the efficacy and safety profile of eptinezumab, a new CGRP monoclonal antibody developed for the treatment of migraines. Although it has a rapid onset of action and half-life of 28 days with no notable drug interactions, it is given intravenously, unlike other monoclonal antibodies such as erenumab which does not require office visits and can be self-administered at home. 

Multiple studies have shown that eptinezumab is an effective preventative medication in migraine which starts showing its effect from day 1 of its administration, and maintains a consistent level of efficacy throughout the year at doses 100mg and 300 mg, both in terms of baseline monthly migraine days (MMDs), as well as patient, reported outcome measures. There is evidence suggesting that in terms of health-related quality of life, its effect is more pronounced in patients with a greater number of MMDs at baseline. This efficacy is seen regardless of patient factors such as age, race, gender, migraine duration, baseline MMDs, and concurrent use of triptans or preventive medication. It is safe to administer in patients with type 1 diabetes or obesity and is effective even in patients with migraine and concurrent medication overuse headaches. Because of its rapid onset of action, it was found to be effective at reducing time to headache pain freedom during acute migraine attacks as well.

Adverse effects are few and mostly mild in intensity. Nasopharyngitis and hypersensitivity are the most common treatment-emergent adverse events. While it is associated with the development of anti-eptinezumab antibodies in a significant number of patients, the clinical significance of this is not yet known, although studies so far have not found any impact of these antibodies on the overall safety profile. Further studies need to be conducted over a longer timeframe as well as in different patient populations to get a bigger and clearer picture of the use of eptinezumab. Comparison studies with other anti-CGRP monoclonal antibodies as well as onabotulinum toxin A would also be needed. 
